# The neuronal and molecular basis of quinine-dependent bitter taste signaling in *Drosophila* larvae

**DOI:** 10.3389/fnbeh.2014.00006

**Published:** 2014-01-27

**Authors:** Anthi A. Apostolopoulou, Lorena Mazija, Alexander Wüst, Andreas S. Thum

**Affiliations:** Department of Biology, University of KonstanzKonstanz, Germany

**Keywords:** *Drosophila* larvae, gustation, bitter, single cell, gustatory receptors, feeding, learning and memory

## Abstract

The sensation of bitter substances can alert an animal that a specific type of food is harmful and should not be consumed. However, not all bitter compounds are equally toxic and some may even be beneficial in certain contexts. Thus, taste systems in general may have a broader range of functions than just in alerting the animal. In this study we investigate bitter sensing and processing in *Drosophila* larvae using quinine, a substance perceived by humans as bitter. We show that behavioral choice, feeding, survival, and associative olfactory learning are all directly affected by quinine. On the cellular level, we show that 12 gustatory sensory receptor neurons that express both GR66a and GR33a are required for quinine-dependent choice and feeding behavior. Interestingly, these neurons are not necessary for quinine-dependent survival or associative learning. On the molecular receptor gene level, the GR33a receptor, but not GR66a, is required for quinine-dependent choice behavior. A screen for gustatory sensory receptor neurons that trigger quinine-dependent choice behavior revealed that a single GR97a receptor gene expressing neuron located in the peripheral terminal sense organ is partially necessary and sufficient. For the first time, we show that the elementary chemosensory system of the *Drosophila* larva can serve as a simple model to understand the neuronal basis of taste information processing on the single cell level with respect to different behavioral outputs.

## Introduction

The sense of taste is the initial evaluation step that determines food quality and is critical for food acceptance or rejection. The bitter taste of a substance alerts an animal not to ingest potentially harmful substances. A well-known bitter substance for humans is quinine, extracted from the bark of the cinchona tree (Scragg and Allan, 1987; Wernsdorfer, 1987; White, 1987). Interestingly, larvae of the fruit fly *Drosophila* avoid quinine and reduce feeding on substrates that contain it (El-Keredy et al., 2012).

*Drosophila* larvae are a powerful experimental system for deciphering information at the single-neuron level, from peripheral sensory organs to higher brain centers, because of the simplicity of its neuronal circuitry, their non-redundant cellular organization, and their genetic tractability (Colomb et al., 2007; Louis et al., 2008; Keene et al., 2011; Kwon et al., 2011). This is illustrated by multiple studies that characterized the larval olfactory system at a fine scale (Ramaekers et al., 2005; Gerber and Stocker, 2007; Masuda-Nakagawa et al., 2009; Selcho et al., 2009; Pauls et al., 2010; Schleyer et al., 2011; Thum et al., 2011). Here, we expand this approach in the gustatory system to obtain a first functional understanding of the molecular and neuronal basis of bitter sensing.

Specific aspects of the gustatory system of the *Drosophila* larva were analyzed in a number of studies (Oppliger et al., 2000; Heimbeck et al., 2001; Wu et al., 2005; Bader et al., 2007; Colomb et al., 2007; Kwon et al., 2011; Mishra et al., 2013). The gustatory apparatus consists of three major external sense organs on the larval head and four internal sense organs located along the pharynx (Singh and Singh, 1984; Python and Stocker, 2002; Gendre et al., 2004). The external organs include the dorsal (DO), terminal (TO), and ventral organs (VO). The internal organs include the dorsal (DPS), posterior (PPS), ventral pharyngeal (VPS) sense organ, and dorsal pharyngeal organ (DPO) (Singh and Singh, 1984; Python and Stocker, 2002; Gendre et al., 2004). Gustatory receptor neurons (GRNs) project from these peripheral and internal sensory organs via four distinct nerves (maxillary, antennal, labral, and labial nerve) to the subesophageal ganglion (SOG) in the central nervous system (Singh and Singh, 1984; Python and Stocker, 2002; Gendre et al., 2004).

There are about 120 sensory neurons located in the anterior part of the larvae and about 90 of them are likely to have gustatory functions. The other 30 neurons are olfactory receptor (21 ORN), temperature sensitive, mechanosensory, and neurons of unknown identity (Python and Stocker, 2002; Fishilevich et al., 2005; Kreher et al., 2005). In *Drosophila* and other insects, GRNs usually respond to water, sugar, low salt, or high salt concentrations. Interestingly, bitter deterrent compounds (e.g., quinine) also activate a subset of high salt-responding neurons (Ebbs and Amrein, 2007; Vosshall and Stocker, 2007; Cobb et al., 2009).

In *Drosophila*, proteins encoded by four different gene families were identified that sense water, sugar, salt, and bitter quality. These include: (i) transient receptor potential channels (TRP), (ii) sodium channels of the *pickpocket* gene family (PPK), (iii) chemosensory ionotropic receptors (IRs), and (iv) seven transmembrane gustatory receptors (GR) that are related to odorant receptors (Chyb et al., 2003; Liu et al., 2003; Thorne et al., 2004; Al-Anzi et al., 2006; Moon et al., 2006; Dahanukar et al., 2007; Benton et al., 2009; Cameron et al., 2010; Weiss et al., 2011; Miyamoto et al., 2012; Zhang et al., 2013). The GR family includes 60 members that are predicted to encode 68 seven-transmembrane receptors through alternative splicing (Scott et al., 2001; Kwon et al., 2011). Several studies demonstrated that individual GR genes are involved in sensing bitter compounds, either as a bitter co-receptor (GR33a) (Moon et al., 2009), or through a specific binding of bitter substances like caffeine (GR66a and GR93a) (Moon et al., 2006; Lee et al., 2009). A similar role for the GR genes was suggested to occur in larvae, although no behavioral studies have focused on the neuronal substrates of bitter sensation (Kwon et al., 2011).

Kwon et al. (2011) anatomically analyzed the larval expression patterns of a set of GAL4 lines for all of the 60 GR genes (Kwon et al., 2011). These lines potentially reflect the endogenous expression of each GR and may allow the establishment of a receptor-to-neuron map. Taken together, 39 of the 68 GRs are expressed at the larval stage in mostly different combinations in at least 16 neurons of the DO, TO, PPS, DPS, and VPS (Kwon et al., 2011). Furthermore, GR66a and GR33a are potentially co-expressed in six GRNs of the external and six GRNs of the internal sensory organs, thereby anatomically and molecularly defining neurons that might be involved in bitter sensing (Kwon et al., 2011). However, the GR66a expression pattern is different from the results of an earlier anatomical study that was based on different Gr66a GAL4 lines (Colomb et al., 2007). Some of the GR GAL4 lines seem to be expressed only in a single GRN (Kwon et al., 2011), allowing for a functional analysis of the bitter taste-induced behaviors up to the single cell level.

In this study, we investigated the behavioral, molecular, and neuronal basis of quinine sensing and processing in *Drosophila* larvae. We show that quinine affects four different larval behaviors (choice, feeding, survival, and associative olfactory learning). We demonstrate that neuronal signaling in only 12 GR66a- and GR33a-positive GRNs is required for quinine-dependent choice behavior and quinine-dependent feeding, but is dispensable for quinine-dependent survival and quinine-reinforced associative olfactory learning. Additionally, we show that the GR33a receptor gene, but not the GR66a receptor gene, is required for quinine-dependent choice behavior. Finally, we identify a single GR97a-positive gustatory neuron in the TO that is necessary and sufficient for quinine-dependent choice behavior. Taken together, we conclude that the perception of quinine is organized by different sensory neurons with respect to specific behaviors. For quinine-dependent choice behavior, the distal group of the TO is important, mainly due to a single neuron that co-expresses the receptor genes GR66a, GR33a, GR57a, and GR97a.

## Materials and methods

### Fly strains

Fly strains were raised on standard *Drosophila* medium at 25°C. All GR-GAL4s, UAS-VR1, UAS-hid,rpr, and UAS-mCD8::GFP stocks were kindly provided by the Carlson, Scott, Sprecher, and Tanimoto lab, respectively. All other strains were obtained from the Bloomington Stock Center. For heterozygous controls, *w1118* was used as a control genotype. *w1118* was also used as an appropriate control in combination with GR33a and GR66a receptor mutants.

For all behavioral experiments, flies were transferred to new vials and allowed to lay eggs for 2 days. The experiments were performed 5 or 6 days after egg laying. Only feeding stage larvae were used, in groups of about 30 animals.

### Choice behavior

Petri dishes were filled with 2.5% (w/ml) agarose solution (agarose in ddH_2_O heated up in a microwave). After cooling down the agarose solution was subsequently removed from the one half of the plate. This half was then filled with the 2.5% (w/ml) agarose-quinine mixture (quinine hemisulfate; Sigma Aldrich; Q1250). The concentration of quinine used varied as described in the results. During the choice assay the larvae were placed in the middle of the plate along the vertical axis and were left to move freely for 5 min. After this time was up, the larvae on the quinine side, on the pure agarose side and in the middle were counted. As a middle zone we define a 1 cm middle zone in the middle of the plate where the larvae were placed at the beginning of the experiment. The Preference Index for each measurement was calculated as follows:

Preference Index=(#quinine side−#pure agarose side)/#total

Negative Preference Indices indicate avoidance behavior toward quinine.

### Feeding

Petri dishes used for the control groups were filled with a solution of 1% (w/ml) agarose and 2% (w/ml) indigo carmin (Sigma Aldrich cat. no.: 73436). Petri dishes used for experimental groups were filled with a solution of 1% (w/ml) agarose, 2% (w/ml) indigo carmin and quinine at various concentrations (please refer to the results). During the feeding assay larvae of all groups were allowed to feed on dishes for 30 min, they were then washed in tap water and homogenized in 500 μ l of 1 M ascorbic acid solution (Sigma Aldrich cat. no.: A7506). The homogenate was centrifuged for 5 min at 13,400 rpm. The supernatant was filtered using a syringe filter (millipore, 5-μm pores) into a new Eppendorf cup and then centrifuged again for 5 min at 13,400 rpm. 100 μl of the supernatant was loaded on a 96-well plate (Hartenstein, Würzburg, Germany). The absorbance at 610 nm of each well mixture was measured using an Epoch spectrophotometer (BioTek, Bad Friedrichshall, Germany). The final absorbance of each single measurement was calculated by deducting the mean absorbance of the blank control (1 M ascorbic acid) from the absorbance of the relative mixture.

$$\begin{array}{l} \text{Absorbance}=\text{absorbance of the mixture}\\ \;-\text{absorbance of the blank control} \end{array}$$

### Survival

Vials used for the control groups were filled with 1% (w/ml) agarose solution and vials used for the experimental groups were filled with 1% (w/ml) agarose and quinine at various concentrations (as described in the Results). Twelve wild-type 1st instar larvae were placed in each and left at 25°C. The number of larvae that were alive was counted each day for 7 consecutive days. Drops of tap water were occasionally added to the vials to prevent larvae from dehydrating. The relative survival of the larvae in each vial was calculated every day by dividing the number of the living larvae on this day with the total number of larvae on day 1.

$$\begin{array}{l} \text{Relative Survival}=\#\text{living larvae on a specific day}/\\ \;\#\text{total larvae on day 1} \end{array}$$

### Associative olfactory learning

For the learning experiments Petri dishes, filled with either 1% (w/ml) agarose solution or 1% (w/ml) agarose and 6 mM Quinine mixture, were used. As olfactory stimuli, 10 μl amyl acetate (AM, Fluka cat. no.: 46022; diluted 1:50 in paraffin oil, Fluka cat. no.: 76235) and 3-octanol (OCT, undiluted; Fluca cat. no.: 74850) were used. The odorants were loaded into custom-made Teflon containers (4.5-mm diameter) with perforated lids as described in Gerber and Stocker (2007). During training a first group of 30 animals were exposed to AM (AM+) while crawling on an agarose medium containing quinine as a negative reinforcer. After 5 min, larvae were transferred to a fresh Petri dish in which they were allowed to crawl on pure agarose medium for 5 min this time being simultaneously exposed to OCT (OCT). A second group of larvae received the reciprocal training (OCT+, AM). After three training cycles, larvae were transferred onto test plates on which AM and OCT were presented on opposite sides. After 3 min, individuals were counted on the AM side (#AM), the OCT side (#OCT), and in a neutral zone. A preference index for each training group is calculated by subtracting the number of larvae on the OCT side from the number of larvae on the AM side and dividing by the total number of counted individuals.

$${\text{Pref}}_{\text{AM+/OCT}}=(\#\text{AM}-\#\text{OCT})/\#\text{total}$$

$${\text{Pref}}_{\text{OCT+/AM}}=(\#\text{AM}-\#\text{OCT})/\#\text{total}$$

A Performance Index (PI) is calculated from the Preference Indices of the two reciprocally trained groups as follows:

$$\text{PI}=({\text{Pref}}_{\text{AM+/OCT}}-{\text{Pref}}_{\text{OCT+/AM}})/2$$

Negative PIs represent aversive quinine-induced learning.

### Artificial activation of the neurons that process quinine sensing

A modified version of the mammalian capsaicin receptor was genetically expressed in different sets of GRNs in the experimental larvae (Wang et al., 2004). Petri dishes were filled with 2.5% agarose solutions on one half and 2.5% agarose and 50 μ M capsaicin mixture on the other half. A capsaicin choice behavior assay was performed and a capsaicin Preference Index was calculated in a similar way as described for the quinine choice behavior experiments. Please note that the capsaicin concentration used was 50 μ M, a concentration at which control larvae do not show a behavioral response. On the other hand, larvae which express the capsaicin receptor in specific GRNs have these neurons artificially activated in the presence of capsaicin. Thus, by studying the behavioral response of these larvae to capsaicin one can get information on the innate function of the activated cells.

### Anatomical analysis

Third instar larvae were dissected in phosphate-buffered saline (PBS). The brains or larval heads were fixed in 3.7% formaldehyde (Merck, Darmstadt) in PBS for 30 min and subsequently washed seven times in PBT (PBS with 3% Triton-X 100, Sigma-Aldrich, St. Louis, MO). Then they were added in 5% normal goat serum (Vector Laboratories, Burlingame, CA) in PBT for 2 h to block unspecific binding and the first antibodies were applied for 2 days at 4°C. Samples were washed six times with PBT and the secondary antibodies were applied for 2 days at 4°C. Finally, samples were washed eight times with PBT, they were mounted in Vectashield (Vector Laboratories) between two cover slips and stored at 4°C in darkness.

For the SOG staining, anti-GFP [Anti-GFP Rabbit, polyclonal serum, A6455, Molecular Probes, (Eugene, OR), 1:1000] was used to label the GRs expression, anti-ChAT [ChAT4B1 Mouse, monoclonal ChAT4B1, DSHB (Iowa City, IA)1:100] was used to label the neuropile, and anti-FasII [1D4 anti-Fasciclin II Mouse, monoclonal, 1D4, DSHB (Iowa City, IA) 1:50] was used to label the axonal tracts. IgG Alexa Fluor 488 (goat anti-rabbit IgG Alexa Fluor 488, A11008; Molecular Probes, 1:200) and IgG Alexa Fluor 647 (goat anti-mouse IgG Alexa Fluor 647 A21236; Molecular Probes, 1:200) were used as secondary antibodies.

For the terminal organ (TO) level staining, anti-GFP [Anti-GFP Rabbit, polyclonal serum, A6455, Molecular Probes, (Eugene, OR), 1:1000] was used to label the GRs expression. Anti-elav [Anti-elav mouse, DHSB (Iowa City, IA), 1:100] was used as a counterstaining to visualize all neurons within the ganglion individually. IgG Alexa Fluor 488 (goat anti-rabbit IgG Alexa Fluor 488, A11008; Molecular Probes, 1:200) was used as secondary antibody. For anti-elav IgG Alexa Fluor 647 (goat anti-mouse IgG Alexa Fluor 647 A21236; Molecular Probes, 1:200) was used as secondary antibody.

Images were taken with a Zeiss LSM510 confocal microscope with a ×25 oil objective. The resulting image stacks were projected and analyzed with Image-J (NIH) software. Contrast and brightness adjustment as well as rotation and organization of images were performed in Photoshop (Adobe Systems Inc., San José, CA).

### Statistical analysis

Kruskal–Wallis test followed by Wilcoxon rank sum test and Holm–Bonferroni correction was used for multiple comparisons. Wilcoxon signed ranked test was used to compare one group against chance level. Statistical analysis was performed with R version 2.14.0 and Windows Excel 2010. The behavioral data were presented as box plots. The middle line within the box shows the median, the box boundaries refer to the 25 and 75% quantiles, and the whiskers represent the 10 and 90% quantiles. Small circles indicate outliers. Asterisks shown in the figures indicate significance levels: n.s., for *p* > 0.05, ^*^ for *p* < 0.05, ^**^ for *p* < 0.01, and ^***^ for *p* < 0.001.

For the survival experiments 15 vials, each of which contained 12 larvae, were used per experimental group. Thus, in total 180 larvae were used per experimental group for the statistical analysis. Kaplan–Meier survival curves were plotted for the representation of the data. Log-rank tests including pair-wise comparisons were performed to detect an overall difference among the curves. To detect differences between two groups for single days (for days 3, 4, and 5), proportion tests were performed, comparing the number of survivors since day 0 between groups.

## Results

### Quinine affects larval choice behavior, feeding, survival, and learning

To address the effect of quinine in different larval behaviors, we assessed the naive behavior of wild type CantonS larvae for quinine-dependent choice behavior, quinine-dependent feeding, survival on quinine, and quinine-reinforced associative olfactory learning [a detailed description of the methods is also given in Rohwedder et al. (2012)].

**Figure 1:**
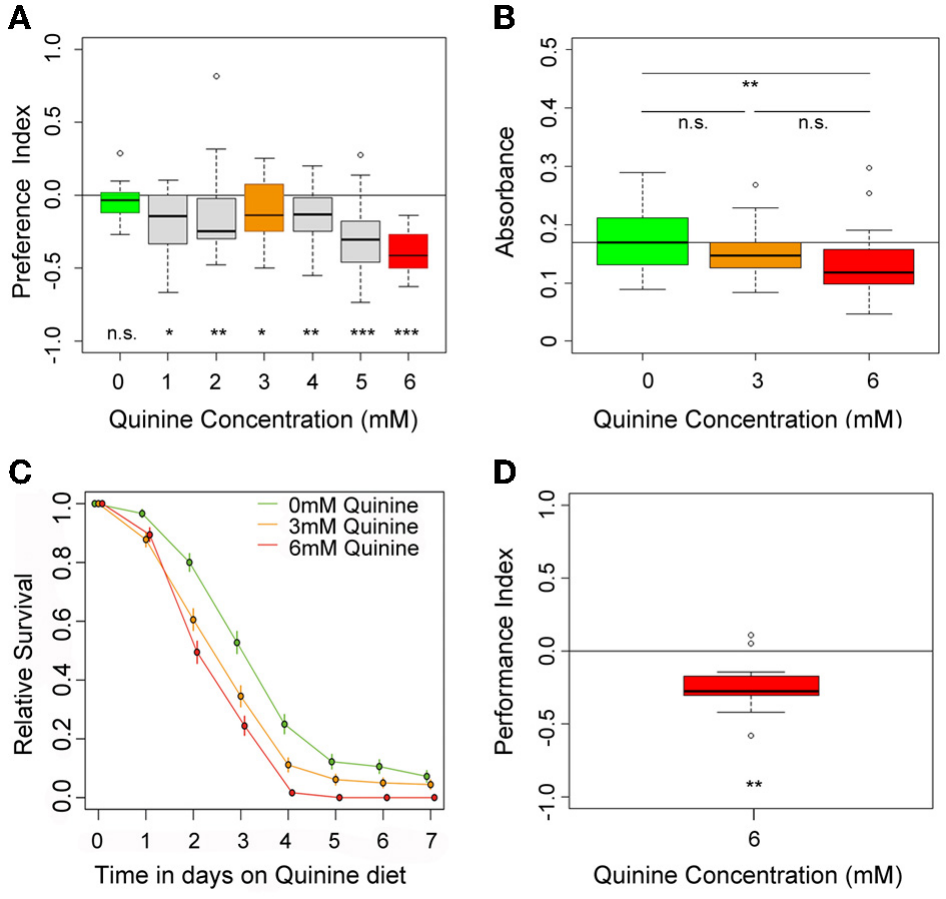
**Quinine affects larval choice behavior, feeding, survival, and associative olfactory learning. (A)** Shows the dose-response curve for larval choice behavior for quinine concentrations between 0 (green) and 6 mM (red). Larvae significantly avoid quinine concentrations from 1 to 6 mM. **(B)** Depicts relative feeding on quinine. Only if 6 mM (red) is mixed into the substrate, larvae reduce feeding significantly with respect to baseline feeding at 0 mM quinine (indicated by the line). A lower concentration of 3 mM, quinine (orange) does not significantly reduce larval food intake. **(C)** Larval survival on agarose-quinine mixture diet depends on the quinine concentration (Kaplan–Meier survival curves). 3 mM quinine reduces larval survival compared to a pure agarose diet. The effect was even stronger for 6 mM quinine as more larvae died during the experiment. **(D)** 6 mM (red) quinine reinforces immediate negative olfactory associative learning when paired with a particular odor. Sample size for each box plot is *n* > 12. Differences against random distribution are given at the bottom of each panel. Differences between groups are presented above the related box-plots (n.s., non-significant *p* > 0.05, ^*^p < 0.05, ^**^p < 0.01, or ^***^p < 0.001). Small circles indicate outliers.

To test for quinine-dependent choice behavior, naive larvae were allowed for 5 min to choose between pure agarose and agarose containing quinine (El-Keredy et al., 2012; Rohwedder et al., 2012). Quinine concentrations ranged from 0 to 6 mM (Figure 1A), the latter being the highest soluble quinine concentration dissoluble in 2.5% agarose. In accordance with previous data (El-Keredy et al., 2012), we found that larvae avoid quinine in a dose-dependent manner (Figure 1A). The side of the Petri dish containing quinine was avoided by larvae, even for low quinine concentrations of 1 mM (*p* < 0.05 compared to random distribution). However, larval choice behavior was more pronounced with increasing quinine concentrations. The strongest avoidance was obtained when 6 mM quinine was present (*p* < 0.001 compared to random distribution).

To test whether quinine affects larval feeding, naive larvae were allowed to feed on 0, 3, or 6 mM quinine-agarose mixtures for 30 min. Afterwards, their food intake was quantified for each mixture with respect to baseline feeding. Consistent with recently published data (El-Keredy et al., 2012), we found that feeding on quinine-agarose mixtures was decreased when compared to feeding on pure agarose (Figure 1B). By increasing the quinine concentration from 0 to 3 mM, the relative feeding did not change (*p* > 0.05). However, relative feeding on 6 mM quinine-agarose mixture was significantly decreased compared to 0 mM baseline feeding (*p* < 0.01).

Next we investigated whether quinine affects larval survival. First instar larvae were placed in vials containing 0, 3, or 6 mM quinine-agarose mixtures as sole food source, and the relative survival was quantified each day for seven consecutive days. Survival differed significantly among treatments (log-rank test, *p* < 0.001). For both quinine concentrations used our data suggested that survival was reduced compared to survival on pure agarose at least from day 3 onwards (proportion tests *p* < 0.001; Figure 1C). The effect of quinine on larval survival was even stronger for 6 mM compared to 3 mM (proportion tests, *p* < 0.05 on day 3; *p* < 0.05 on day 4; *p* < 0.001 on day 5).

Finally, we used a well-established assay to analyze quinine-reinforced associative olfactory learning (Hendel et al., 2005; Schleyer et al., 2011; El-Keredy et al., 2012). Pairing an odor stimulus with quinine induced an aversive association (Figure 1D; *p* < 0.01). Thus, for *Drosophila* larvae, quinine serves as a negative, punishing reinforcer in associative olfactory learning.

### GR33a and GR66a neuronal signaling is required for quinine-dependent choice behavior and feeding but not for survival and learning

**Figure 2:**
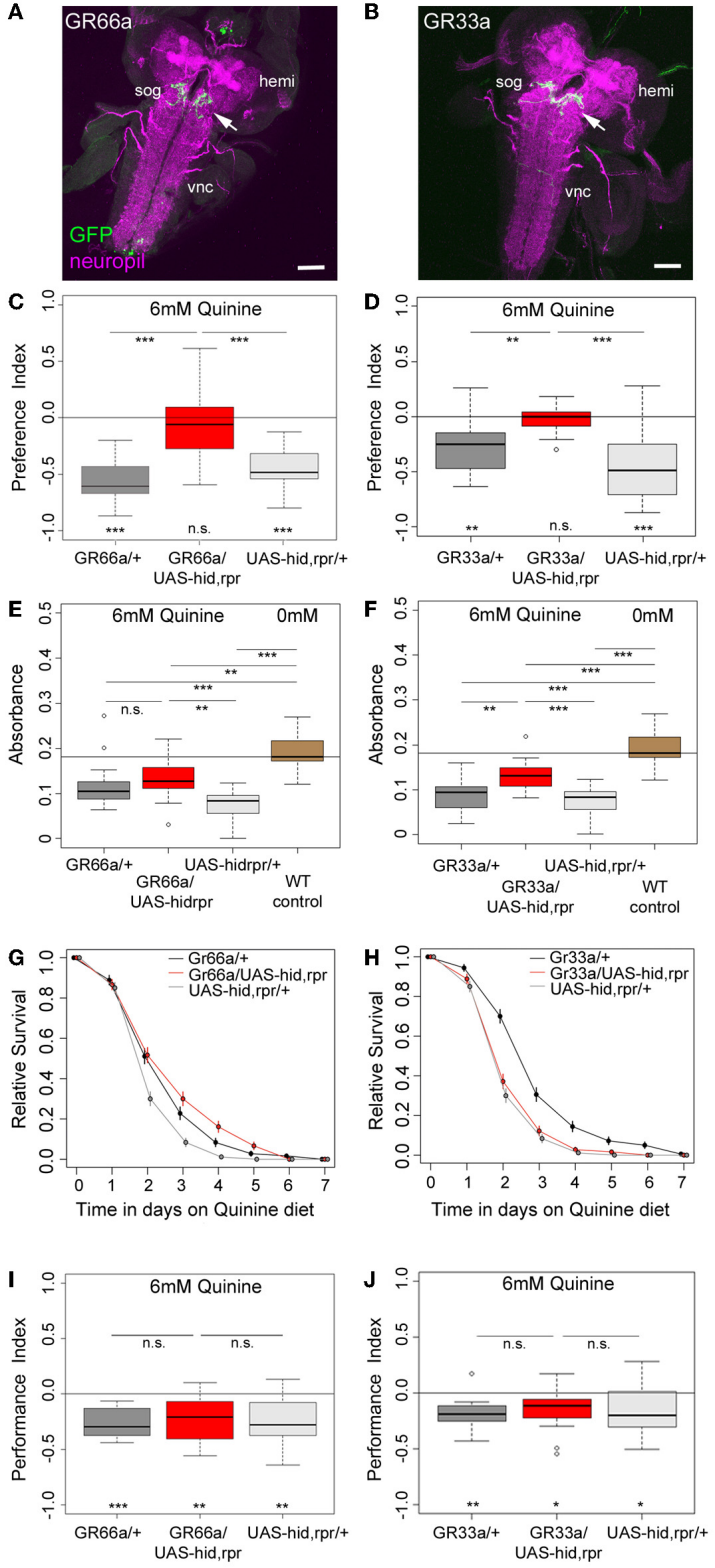
**GR66a and GR33a neuronal signaling is required for quinine-dependent choice behavior and feeding, but dispensable for survival and associative olfactory learning. (A,B)** Show frontal views of CNS projections of GR66a-GAL4 and GR33a-GAL4 lines crossed with UAS-mCD8::GFP. To visualize the expression patterns, the CNS was stained with anti-GFP (green) and anti-ChAT; anti-FasII (magenta). In both lines, GRNs innervate the SOG in a similar way (arrows); sog, subesophageal ganglion; vnc, ventral nerve cord; hemi, brain hemisphere. **(C,D)** Genetically ablating the GRNs covered by GR66a-GAL4 or GR33a-GAL4 expression completely diminishes larval choice behavior for 6 mM quinine. Both experimental larvae behave significantly different from genetic controls [*p* < 0.001 in **(C)** and *p* < 0.01 and *p* < 0.001 in **(D)**] and do not show any aversive choice behavior (n.s.). **(E,F)** Genetically ablating GRNs covered by GR66a-GAL4 or GR33a-GAL4 expression partially increases feeding behavior on a substrate containing 6 mM quinine. GR66a/UAS-hid,rpr **(E)** and GR33a/UAS-hid,rpr **(F)** experimental larvae feed more than control larvae on a substrate containing 6 mM quinine [n.s. and *p* < 0.01 in **(E)**; *p* < 0.01 and *p* < 0.001 in **(F)**]. Both experimental groups do not feed on baseline level, i.e., wild type control larvae on pure agarose substrate [*p* < 0.01 in **(E)**; *p* < 0.001 in **(F)**]. **(G,H)** Larval survival is plotted as Kaplan–Meier survival curves; genetically ablating the GRNs covered by GR66a-GAL4 or GR33a-GAL4 expression does not reduce larval survival on a 6 mM quinine agarose mixture diet. Neither GR66a/UAS-hid,rpr **(G)** nor GR33a/UAS-hid,rpr **(H)** experimental larvae live longer than both control groups [log-rank test, n.s. for the GR66a-GAL4 control, but log-rank test, *p* < 0.001 for the UAS-hid,rpr control in **(G)**; log-rank test, n.s. for the UAS-hid,rpr control in **(H)**, but log-rank test *p* < 0.001 for the GR66a-GAL4 control also in **(H)**]. **(I,J)** Genetically ablating GRNs covered by GR66a-GAL4 or GR33a-GAL4 expression does not affect associative olfactory learning reinforced by 6 mM of quinine. GR66a/UAS-hid,rpr **(I)** and GR33a/UAS-hid,rpr **(J)** experimental larvae perform on the same level as respective control groups (n.s.) in an assay testing quinine-reinforced associative olfactory learning. Sample size for each box plot is *n* > 12. Differences against zero are given at the bottom of each panel. Differences between experimental groups are depicted above the respective box plots (n.s., non-significant *p* > 0.05, ^*^p < 0.05, ^**^p < 0.01, or ^***^p < 0.001). Scale bars: 50 μm. Small circles indicate outliers.

**Figure 3:**
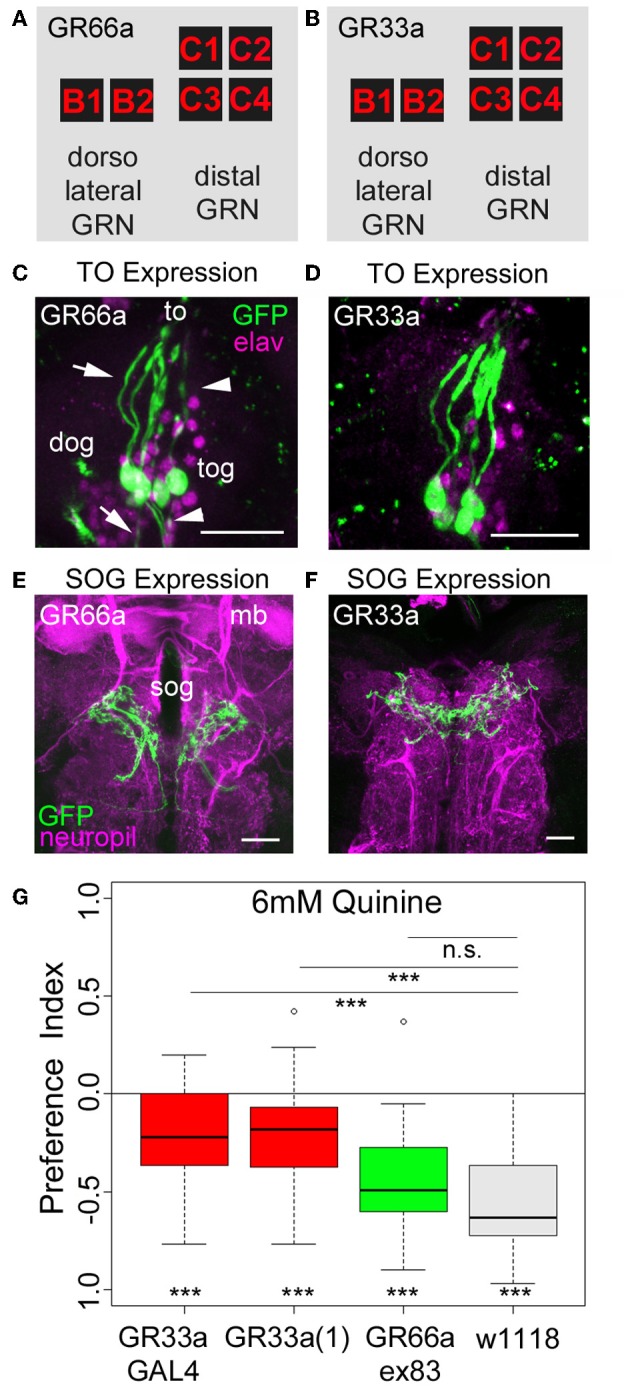
**Gr33a receptors but not Gr66a receptors are required for quinine avoidance. (A,B)** GR66a-GAL4 and GR33a-GAL4 are expressed in the same set of six GRNs of the TO, schematically represented for GR66a-GAL4 **(A)** and GR33a-GAL4 **(B)**. Two of the neurons belong to the dorsolateral group, called B1 and B2, and the other four to the distal group, called C1–C4. This suggests that both receptors are co-expressed in the same set of bitter sensing GRNs. **(C,D)** The GFP expression for GR66a-GAL4 **(C)** and GR33a-GAL4 **(D)** crossed to UAS-mCD8::GFP is shown in the terminal organ (to). The two GRNs B1 and B2 of the dorsolateral group have their cell bodies located within the dorsal organ ganglion (dog; arrow). The four GRNs C1–C4 of the distal group have their cell bodies in the terminal organ ganglion (tog; arrowhead). Anti-GFP (green) and anti-elav (magenta) antibodies were used to visualize the specimens. **(E,F)** In a frontal view part of the central projection of GR66a-GAL4 **(E)** and GR33a-GAL4 **(F)** is shown in the SOG (sog). The projections were traced by crossing the two GAL4 lines with UAS-mCD8::GFP. GFP is visualized by anti-GFP antibody staining (green) and the neuropil by anti-ChAT; anti-FasII antibody staining (magenta). **(G)** When testing different mutants for the GR33a and GR66a receptor, only mutants affecting GR33a receptor function [GR33a(1) and GR33aGAL4] show a reduced choice behavior in the presence of 6 mM of quinine, compared to an appropriate w1118 control group (*p* < 0.001). A mutant that abolishes GR66a receptor function (GR66aex83) does not change choice behavior (n.s.) compared to the appropriate w1118 control. Sample size for each box plot is *n* > 12. Differences against zero are given at the bottom of each panel. Differences between experimental groups are depicted above the respective box plots (n.s., non-significant *p* > 0.05 and ^***^p < 0.001). mb, mushroom body; scale bars: 25 μm in **(C)** and **(D)**; 20 μm in **(E)** and **(F)**. Small circles indicate outliers.

Recently, Kwon et al. (2011) analyzed the cellular organization of all GR genes by using a comprehensive set of GR-GAL4 driver lines of the GAL4/UAS expression system (Brand and Perrimon, 1993). They demonstrated that GR66a (suggested to be involved in larval bitter sensing) and GR33a are co-expressed in six neurons of the TO and likely in six neurons of the pharyngeal organs of the larval head (Kwon et al., 2011). However, these results are in contrast to an earlier study that showed that another GR66a-GAL4 line is only expressed in about three to four neurons of the external sensory organs (Colomb et al., 2007). By expressing UAS-mCD8::GFP via the initially published GR66a and the newly published GR33a driver lines, we were able to reproduce in whole mount preparations the recently described expression patterns, both in the periphery and within the SOG (Figures 2A,B, 3A–F) (Kwon et al., 2011).

To investigate whether GR66a and GR33a neuronal signaling is necessary for quinine-dependent choice behavior, feeding, survival, and associative olfactory learning, we genetically ablated these GRNs by co-expression of *hid* and *reaper*. Ectopic expression of these genes induces apoptosis through caspase activation (White et al., 1996; Kurada and White, 1998; Selcho et al., 2009, 2012).

When GR66a neurons are ablated, experimental larvae do not show any avoidance of 6 mM quinine over pure agarose (Figure 2C, *p* > 0.05); in contrast, control larvae show robust avoidance (*p* < 0.001 for both controls). Similarly, larvae with ablated GR33a neurons fail to show any avoidance of 6 mM quinine (Figure 2D, *p* > 0.05), whereas the controls show strong avoidance (*p* < 0.01 for the GAL4 control and *p* < 0.001 for the UAS control).

In the feeding assay, feeding of control larvae on 6 mM quinine was significantly reduced compared to wild type larval feeding on 0 mM quinine (Figures 2E,F; *p* < 0.001 for all comparisons with WT control larvae measured at 0 mM quinine). When GR66a-GAL4-positive GRNs are ablated, larvae show increased relative feeding on 6 mM quinine as compared to the UAS control (*p* < 0.01) but do not differ from the GAL4 control (Figure 2E; *p* > 0.05). When GR33a-GAL4-positive GRNs are ablated, larvae show increased relative feeding as compared to both controls (Figure 2F; *p* < 0.01 compared to the GAL4 control and *p* < 0.001 compared to the UAS control). Although feeding after GR66a or GR33a neurons ablation is increased, it does not reach the feeding levels of wild type larvae on 0 mM quinine (*p* < 0.01 when GR66a neurons are ablated *p* < 0.001 when the GR33a neurons are ablated).

When Gr66a neurons are ablated, the overall survival of the larvae on quinine was not observed to be significantly different from the overall survival of the GAL4 control (log-rank test, *p* = 0.22). However, survival was better compared to the overall survival of the UAS-hid,rpr control (Figure 2G, log-rank test *p* < 0.001). When Gr33a neurons are ablated, the overall survival of the larvae on quinine is reduced compared to the overall quinine survival of the GAL4 control (Figure 2H, log-rank test *p* < 0.001) but was not found to differ from the overall quinine survival of the UAS-hid,rpr control (log-rank test, *p* > 0.05). Thus, taken both cases together, we do not find a consistent increase or decrease of the survival rate of the experimental groups in comparison with their genetic controls.

Next, we tested if the described set of GRNs is necessary for quinine-reinforced associative olfactory learning. Strikingly, ablation of the GR66a-GAL4-positive GRNs still allows experimental larvae to form olfactory associations reinforced by quinine (Figure 2I; *p* < 0.01). They even perform on a level comparable to control larvae (Figure 2I; *p* > 0.05 from both controls). Similarly, after GR33a GRN ablation, experimental larvae are still able to establish quinine-reinforced odor associations (Figure 2J; *p* < 0.05). Again the performance does not differ from control groups (Figure 2J; *p* > 0.05 from both controls).

Taken together, the results suggest that GR66a-GAL4- and GR33a-GAL4-positive GRN signaling is required for quinine-dependent choice behavior, is partially required for quinine-dependent feeding behavior, but is dispensable for quinine-dependent survival and associative olfactory learning.

### GR33a receptor gene function but not GR66a receptor gene function is required for quinine-dependent choice behavior

As quinine-dependent choice behavior seems to completely rely on the neuronal output of GR66a-GAL4- and GR33a-GAL4-positive GRNs, we further focused on this particular behavior. First, we asked if only a single receptor gene or both receptor genes co-expressed in these neurons are required for quinine-dependent choice behavior. In adult flies, the GR33a receptor gene is required to elicit a proper electrophysiological response of GRNs to quinine as well as to express an appropriate quinine avoidance behavior (Moon et al., 2009). In contrast, the GR66a receptor gene is neither required to elicit a proper electrophysiological response to quinine nor affects adult quinine-dependent avoidance behavior (Moon et al., 2009). To test whether the molecular function of quinine-dependent choice behavior is conserved between the adult and larval stage of *Drosophila*, we used three different mutants that disrupt either GR66a (Gr66aex83) or GR33a [Gr33a-GAL4 and Gr33a(1)] receptor gene function (Moon et al., 2006, 2009). Gr66ex83 mutant larvae showed normal quinine-dependent choice behavior comparable to control larvae (Figure 3G; *p* > 0.05 compared to the control), whereas the Gr33a-GAL4 and Gr33a(1) mutants showed significantly reduced avoidance (Figure 3G; *p* < 0.001 for both mutants). These data suggest that in larvae, similarly to adult flies, quinine-dependent choice behavior requires GR33a receptor gene function and is independent of GR66a receptor gene function.

### A single GRN of the terminal organ is required for proper quinine-dependent choice behavior

As ablation of GR66a-GAL4- and GR33a-GAL4-positive GRNs completely abolishes quinine-dependent choice behavior (Figures 2C,D), we next analyzed the correlation between behavioral function and neuronal circuit on the single-cell level. Kwon et al. (2011) reported, in line with our data presented here, that GR66a-GAL4 and GR33a-GAL4 expression co-localize in two neurons of the dorsolateral group of the TO (neurons B1 and B2), in four neurons of the distal group of the TO (C1–C4) and in six neurons of the pharyngeal organs (see also Figures 3A–F).

**Figure 4:**
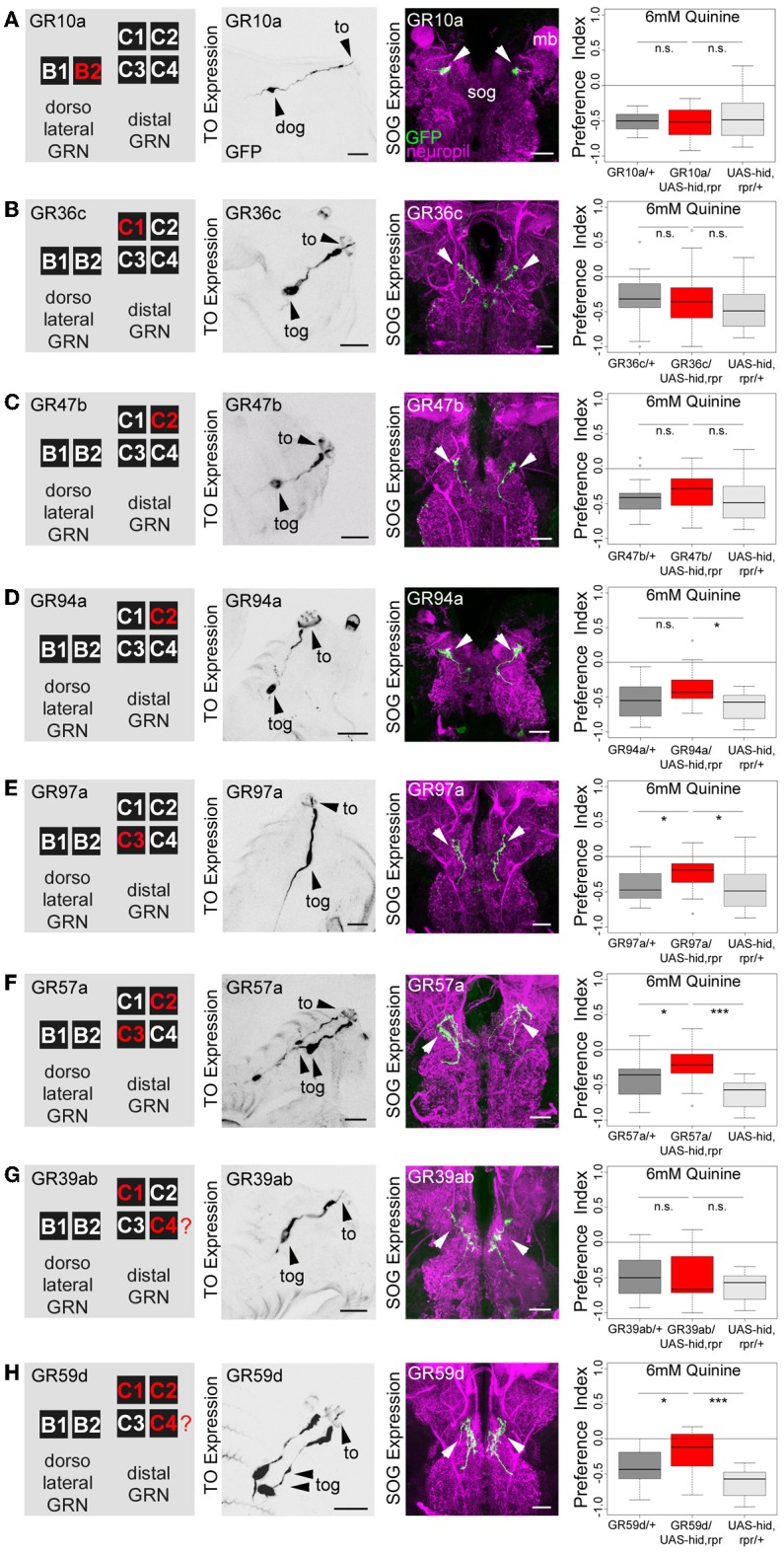
**Single TO neurons—behavior function correlation for quinine-dependent choice behavior**. The first column shows a schematic overview of the respective GRNs included in the expression pattern of each GAL4 line, based on Kwon et al. (2011). The second column illustrates the expression pattern for each GAL4 line crossed with UAS-mCD8::GFP at the level of the TO (arrowheads mark the indicate the innervation of the terminal organ and the position of the respective cell body in the dorsal organ ganglion or terminal organ ganglion). The third column shows in frontal views part of the GRN projections in the SOG for each GAL4 line. These were crossed with UAS-mCD8::GFP and stained by anti-GFP (green) and anti-ChAT, anti-FasII (magenta) antibody staining. The last column shows the quinine-dependent choice behavior for each GAL4 line when crossed to UAS-hid,rpr in order to specifically induce cell death in small sets of GRNs or single GRNs. The analysis includes GR10a-GAL4 **(A)**, GR36c-GAL4 **(B)**, GR47b-GAL4 **(C)**, GR94a-GAL4 **(D)**, GR97a-GAL4 **(E)**,GR57a-GAL4 **(F)**, GR39ab-GAL4 **(G)**, and GR59d-GAL4 **(H)**. Ablation of the B2, C1, C2 neurons alone does not alter quinine-induced choice behavior [n.s. for each experimental genotype compared to both control groups in **(A–D)**; except for the UAS-hid,rpr control in **(D)**; *p* < 0.05]. However, ablation of C3 only (**E**; *p* < 0.05 to respective controls) or in combination with C2 (**F**; *p* < 0.05 and *p* < 0.001 to respective controls) reduces choice behavior significantly. Ablation of C1, C2, and C4 in combination (in **H**) significantly reduces choice behavior as well (*p* < 0.05 and *p* < 0.001 to respective controls); expression within GRNs other than in the TO is not analyzed here. For further details see also Kwon et al. (2011). The identity of the C4 neuron (in **G,H**) as proposed by Kwon et al. (2011) was not verifiable in our specimens (indicated by a “?”). Sample size for each box plot is *n* > 12. Differences between experimental groups are depicted above the respective box plots (n.s., non-significant *p* > 0.05, ^*^p < 0.05, and ^***^p < 0.001; Small circles indicate outliers). mb, mushroom body; to, terminal organ; dog, dorsal organ ganglion; tog, terminal organ ganglion; sog, subeosophageal ganglion; scale bars: 20 μm.

The same authors also published a receptor-to-neuron map, in which subsets of the GR66a and GR33a TO neurons are matched with the expression patterns of other GR-GAL4 drivers. From this map, we selected the following GR-GAL4s to analyze the function of each peripheral neuron on the single-cell level: GR10a-GAL4, GR36c-GAL4, GR47b-GAL4, GR94a-GAL4, GR97a-GAL4, GR57a-GAL4, GR39a.b-GAL4, and GR59d-GAL4. The left column in Figure 4 illustrates in which of the GRNs the different drivers express GAL4 (Kwon et al., 2011), namely GR10a-GAL4 in the B2 neuron of the TO dorsolateral group (Figure 4A), GR36c-GAL4 in the C1 neuron of the TO distal group (Figure 4B), GR47b-GAL4 and GR94a-GAL4 in the C2 neuron of the TO distal group (Figures 4C, D), GR97a-GAL4 in the C3 neuron of the TO distal group (Figure 4E), GR57a-GAL4 in the C2 and C3 neuron of the TO distal group (Figure 4F), GR39a.b-GAL4 in the C1 and C4 neuron of the TO distal group (Figure 4G) and GR59d-GAL4 in the C1, C2, and C4 neuron of the TO distal group (Figure 4H). We were able to confirm most of these results by crossing each GR-GAL4 driver to UAS-mCD8::GFP and analyzing the expression in the TO and the SOG (Figures 4A–H, second and third column). However, in GR39a.b-GAL4 and GR59d-GAL4, the C4 neuron is not present in our samples (Figures 4G,H, indicated by a red question mark in the presented scheme).

After the anatomical verification of the GR-GAL4 expression patterns, we next crossed each GR-GAL4 with UAS-hid,rpr to again ablate small sets of GRNs or single GRNs. We then assessed the quinine-dependent choice behavior for each experimental group in order to identify individual neurons necessary for the behavior. Ablation of individual GRNs using GR10a-GAL4 (Figure 4A), GR36c-GAL4 (Figure 4B), GR47b-GAL4 (Figure 4C), and GR94a-GAL4 (Figure 4D) does not alter the larval response to quinine (*p* > 0.05 for all experimental groups when compared to controls, except for GR94a/UAS-hid,rpr compared to UAS-hid,rpr in Figure 4D; *p* < 0.05). Thus, the single neurons B2, C1 and the C2 are not necessary for quinine-dependent choice behavior. On the contrary, ablation of the C3 neuron alone using GR97a-GAL4 (Figure 4E; *p* < 0.05 compared to the GAL4 and UAS control) or in combination with the C2 neuron, by using GR57a-GAL4 (Figure 4F; *p* < 0.05 compared to the GAL4 control and *p* < 0.001 compared to the UAS control) significantly reduced quinine-dependent choice behavior (however not completely). We observe a similar phenotype when all neurons of the TO distal group - except C3 - are ablated, by using GR59d-GAL4 (Figure 4H, *p* < 0.05 compared to the GAL4 control and *p* < 0.001 compared to the UAS control). Taken together, we conclude that the single Gr97a positive GRN C3 of the TO distal group is mainly, however not exclusively, required for quinine-dependent choice behavior.

### Activation of a single GRN of the terminal organ is sufficient to express a proper choice behavior

**Figure 5:**
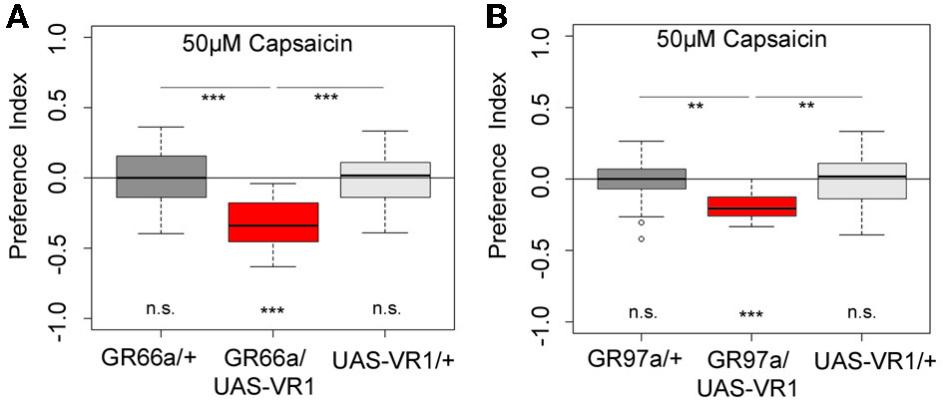
**Artificial activation of small set of GRNs or of the single C3 neuron is sufficient to induce an aversive choice behavior**. Expression of the mammalian vanilloid receptor protein (VR1) allowed to quantify the behavioral relevance of GR66a-GAL4- and GR97a-GAL4-positive neurons after capsaicin-dependent activation. **(A)** Experimental GR66a/UAS-VR1 larvae avoided a 50 μM capsaicin agarose mixture against pure agarose (*p* < 0.001). The behavioral response was significantly different than the appropriate genetic controls (*p* < 0.001 each) that did not avoid capsaicin (n.s.). **(B)** Activation of a single GRN C3 in the TO in GR97a/UAS-VR1 experimental larvae induced an aversive capsaicin-dependent choice behavior (*p* < 0.001) that was significantly different from both genetic controls (*p* < 0.01 for both controls). Thus, activation of the single GRN C3 is sufficient to elicit gustatory-guided choice behavior. Sample size for each box plot is *n* > 12. Differences against zero are given at the bottom of each panel. Differences between experimental groups are depicted above the respective box plots (n.s., non-significant *p* > 0.05, ^**^p < 0.01, and ^***^p < 0.001). Small circles indicate outliers.

Next we wanted to investigate whether artificial activation of all 12 GR66a-GAL4- positive neurons or activation of the single C3 (via GR-97a-GAL4) neuron alone is sufficient to elicit a similar aversive behavioral response. To activate the respective GRNs, we expressed a modified version of the mammalian vanilloid receptor protein (VR1) specific for capsaicin in the two sets of GRNs and assessed the capsaicin-dependent choice behavior (Marella et al., 2006; Colomb et al., 2007). Control larvae that do not express VR1 in GRNs did not respond to capsaicin (Figure 5, *p* > 0.05 for all the controls). However, when Gr66a neurons expressed VR1, larvae strongly avoided capsaicin (Figure 5A, *p* < 0.001). These results are in accordance with the data published by Colomb et al. (2007) and reconfirm that the activation of Gr66a neurons can elicit a repulsive behavior. We then expressed VR1 in the single Gr97a positive C3 cell of the TO. Again, experimental larvae strongly avoided capsaicin (Figure 5B, *p* < 0.001), whereas controls did not show any capsaicin response (*p* > 0.05). Thus, activation of the single Gr97a-positive C3 GRN is sufficient to elicit aversive choice behavior.

## Discussion

### Bitter taste in drosophila

Unlike the olfactory system, in which the one-neuron-one-receptor hypothesis nearly holds true (Fishilevich et al., 2005; Kreher et al., 2005), in the gustatory system, different receptors are co-expressed in partially overlapping neuronal sets. For example, up to 17 GRs are co-expressed in the larval C1 neuron and 29 GRs in a labellar neuron of the adult fly (Kwon et al., 2011; Weiss et al., 2011). Thus, at the receptor level, the gustatory systems of larvae and flies differ significantly from the respective olfactory systems. It was therefore speculated that, in contrast to the discrimination-optimized olfactory system, the taste system provides a hedonic rating of substances into, for example, “non-edible/bitter” vs. “edible/sweet” (Marella et al., 2006; Cobb et al., 2009).

However, recent studies in flies suggest that the gustatory system has a coding capacity that is beyond sensing exclusively bitter or sweet. A study by Weiss et al. (2011) on the bitter taste system of adult *Drosophila* defined four classes of bitter sensing neurons that are diverse in their response profiles. The response of single bitter GRNs to different bitter compounds varied in their study with respect to specificity, temporal dynamics, and onset kinetics (Weiss et al., 2011). Therefore, bitter substances, including quinine, are not uniformly sensed among “bitter neurons.” Instead, a discrimination-optimized gustatory system allows for the distinction between different bitter substances and possibly for the evaluation of different bitter stimuli with respect to particular behaviors. However, the work by Masek and Scott (2010) suggests that flies do not discriminate among different sugars, or among different bitter compounds, based on chemical identity but rather with limited ability based on intensity or palatability when retrieving gustatory memories after taste associative learning. Thus, under these conditions the discriminative capacity of the fly taste system is obviously limited compared to the olfactory system (Masek and Scott, 2010).

Discriminative capacity may also be a property of the simpler larval taste system. For instance, *Drosophila* larvae can distinguish between different sugars like fructose and glucose in a behavioral choice assay (Miyakawa, 1982). Larvae of other insect species also show a higher degree of complexity, as shown by the identification of heterogeneous bitter sensing cells (Glendinning et al., 2002, 2006). This might indicate that the general properties of the gustatory system may be conserved between the larval and adult stage. Hence, due to their reduced cellular complexity and their attractiveness for functional and molecular studies, larvae allow for simpler access to the functional architecture of the taste system, from single cells up to the systems level.

### Sensing and processing of quinine in drosophila larvae—choice behavior

*Drosophila* larvae change their behavior as soon as they contact quinine. They avoid quinine in a choice assay, they reduce feeding on a quinine containing substrate, they die earlier on quinine containing food, and they associate quinine with a simultaneously presented odor (Figure 1) (El-Keredy et al., 2012). Quinine affects larval behaviors negatively and in a dose-dependent manner, as higher amounts of quinine increase choice behavior (Figure 1A) and decrease survival (Figure 1C). Signaling of GR66a and GR33a GRNs is necessary for quinine-dependent choice behavior (Figures 2C,D) and partially for quinine-dependent feeding (Figures 2E,F). Thus, the output of no more than 12 sensory neurons, situated in the TO (two GRNs of the dorsolateral group and four GRNs of the distal group), DPS (two), VPS (two), and PPS (two) is encoding quinine bitter information for these behaviors. The effect for quinine-dependent choice behavior can even be partially traced to the output of a single GR97a-positive C3 neuron (Figures 4, 5).

**Figure 6:**
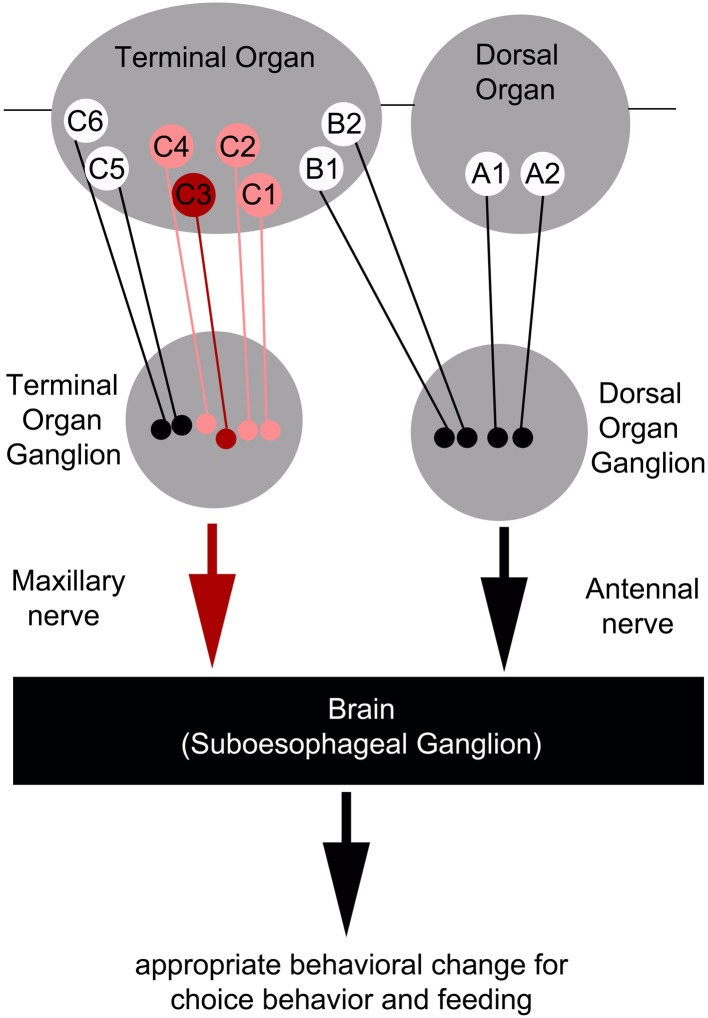
**Schematic overview of the neurons signaling quinine-dependent bitter taste in Drosophila larvae**. According to Kwon et al. (2011), GRs are expressed in only 10 neurons of the two major chemosensory organs of the larva, the dorsal organ (DO) and the terminal organ (TO). The two GRNs of the DO are called A1 and A2 (DO group). Additionally, B1 and B2, which are located in the dorsolateral group of sensilla of the TO, have their cell bodies sitting in the DO ganglion (TO dorsolateral group). C1–C6 located in the distal group of sensilla of the TO have their cell bodies located in the TO ganglion (TO distal group). Bitter quinine taste information affecting larval choice behavior is mediated mainly by the TO neurons C1–C4 (pink) and especially by the single TO neuron C3 (red). Signals reach the subesophageal ganglion via the maxillary nerve. Output of the single C3 neuron is necessary for quinine-dependent avoidance and artificial activation of the C3 neuron is sufficient to elicit aversive choice behavior.

However, as C3 neuron ablation does not cause a full suppression of quinine-dependent choice behavior (Figure 4E), we believe that additional neurons might be involved. Possible candidates are the C1, C2, and C4 neurons of the distal group of the TO, because their elimination also leads to a reduction in choice behavior (Figure 4H). Our data suggest that, for choice behavior, quinine-related sensory information is signaled via the maxillary nerve to the CNS by a maximum of four GRNs belonging to the distal group of TO neurons (Figure 6).

### Sensing and processing of quinine in drosophila larvae—associative olfactory learning

Surprisingly, the same set of 12 GRNs are dispensable for quinine-dependent associative olfactory learning (Figures 2I,J), revealing a different neuronal basis for quinine-dependent punishment sensing and signaling. The neurons involved in the alternative quinine signaling pathways are yet unknown. However, several possibilities can be taken into consideration based on recent findings on sugar reward processing in larvae and flies (Inoshita and Tanimura, 2006; Burke and Waddell, 2011; Miyamoto et al., 2012; Rohwedder et al., 2012; Dus et al., 2013; Gruber et al., 2013; Mishra et al., 2013) These studies suggest that sugar reward is not due to a single-GRN-based signal, but on a more complex set of sensory inputs that integrate different types of sensory information. If negative inputs are similarly encoded, there are several possibilities that might signal punishing reinforcement in larval odor-quinine learning. The following possibilities could occur alone or in combination with GR66a–GR33a GRN signaling. First, an additional set of GRNs may exist that internally measure the bitter quality of the consumed quinine, similar to the internal fructose sensors in flies (Miyamoto et al., 2012; Mishra et al., 2013). Second, feeding on an aversive substrate may measure the osmolarity of the consumed food for an internal evaluation that is important for punishment processing (Gruber et al., 2013). Third, the punishing signal of quinine may not, or only partially, be related to GRN signaling. Instead, it may be an indirect consequence of its harmful effect(s) after consumption (Figure 1C). Indeed, in honeybees it was shown that quinine seems to induce a post-ingestional malaise-like state that retards olfactory learning, induces mortality in a dose-dependent manner, and reduces the behavioral response to the US and CS after appetitive olfactory conditioning (devaluation) (Ayestaran et al., 2010). Therefore, honeybees possess an inherent ability to selectively associate gustatory cues with quinine-dependent malaise. Larvae, similar to honeybees, may also learn to associate the negative effect on their fitness with a given odor. It is not clear how such a cue could be measured and if this process would be quick enough to be turned on and off within the required seconds. Fourth, additional external quinine sensors may exist on the surface of the larvae. Our analysis does not include the ventral organ, a small sensory organ that also contains seven GRNs (Python and Stocker, 2002). Additionally, aversive information might also be perceived by multidendritic neurons that cover the body wall. Such neurons were recently reported to signal negative information for different sensory modalities (Hwang et al., 2007; Xiang et al., 2010; Zhong et al., 2010). Taken together, our data suggest that bitter punishment signaling is organized similarly to reward signaling, as it is based on complex processing of different inputs independent of, or in addition to, GRN signaling (Rohwedder et al., 2012).

### The GR33a receptor is necessary for quinine-dependent choice behavior

The GR66a-GAL4 and GR33a-GAL4 lines target the same GRNs because the genes encoding GR66a and GR33a are co-expressed in the same neurons. Genetically induced apoptosis of these neurons completely abolishes choice behavior (Figures 2C,D). However manipulating neuronal function does not demonstrate receptor function. We therefore, used a set of receptor mutants to show that quinine-dependent choice behavior relies on GR33a, but not GR66a receptor function (Figure 3G). In adults, GR33a receptor function is required for responses to many bitter substances including quinine, caffeine, denatonium, berberine, lobeline, papaverine, and strychnine (Moon et al., 2009). However, GR66a receptor function was shown to be dispensable for quinine-dependent responses in flies (Moon et al., 2006). This suggests conservation of receptor function for these GRs between the two developmental stages. This is striking, as during metamorphosis the GRNs of the larval TO undergo apoptosis and adult external GRNs are formed *de novo* (Gendre et al., 2004).

Furthermore, adult flies and larvae may use GR33a as a bitter co-receptor, comparable to the proposed role of the Orco receptor for the olfactory system (Larsson et al., 2004). A similar role was also suggested for GR66a in flies (Weiss et al., 2011). However, as GR33a-GAL4 and GR66a-GAL4 are potentially the only GRs expressed in a single B1 neuron of the dorsolateral group of the TO (the information is based on GAL4 expression data and may not reflect the endogenous GR gene expression), both receptors cannot exclusively function as co-receptors that require another GR for ligand specificity (Kwon et al., 2011).

### Conclusions from the larval system on drosophila gustation

A comparison of the larval and adult chemosensory systems shows that the former includes larval-specific elements in the periphery and elements shared with the adult system in the CNS (Python and Stocker, 2002; Colomb et al., 2007). This “hybrid” organization is perhaps related to the transformation of the system during metamorphosis, as its sensory components are almost completely replaced (Tissot and Stocker, 2000; Gendre et al., 2004). Thus, any interpretation of data in the larval system has to take into account its special larval design. Nevertheless, the parallels between the larval and adult gustatory systems render larvae a valuable system to comprehensively describe the functional principles of taste sensing. In particular, the larval gustatory system offers a non-redundant organization that can be experimentally interrogated through a combination of high-resolution behavioral analysis and cutting-edge neurogenetic tools for remote-control of the activity of single neurons.

## Author contributions

Anthi A. Apostolopoulou designed and performed the experiments, analyzed the data and wrote the manuscript. Lorena Mazija and Alexander Wüst performed the experiments and analyzed the data. Andreas S. Thum designed the experiments, analyzed the data, and wrote the manuscript.

### Conflict of interest statement

The authors declare that the research was conducted in the absence of any commercial or financial relationships that could be construed as a potential conflict of interest.
